# Case Report: Mixed gonadal dysgenesis with Müllerian remnants mimicking a prostatic utricle in a child with 45,X/46,XY/47,XYY mosaicism

**DOI:** 10.3389/fped.2026.1915927

**Published:** 2026-08-31

**Authors:** Shuai Zhang, Chenying Zhou, Dianyong Liu

**Affiliations:** Department of Pediatric Urology, Dalian Women and Children's Medical Group, Dalian, Liaoning, China

**Keywords:** 45, X/46, XY/47, XYY mosaicism, anti-Müllerian hormone, case report, disorder of sex development, mixed gonadal dysgenesis, Müllerian duct remnants, proximal hypospadias, sex-chromosome mosaicism

## Abstract

**Background:**

Sex-chromosome mosaicism can cause discordant gonadal, ductal, and external genital development. Persistent Müllerian derivatives are classically associated with defects in anti-Müllerian hormone (AMH) production or AMH receptor type 2 (AMHR2) signaling in otherwise normally virilized 46,XY individuals, termed persistent Müllerian duct syndrome (PMDS). In complex mosaicism, however, Müllerian persistence may reflect mixed gonadal dysgenesis (MGD).

**Case presentation:**

A male-reared child presented with marked short stature, severe proximal hypospadias, ventral chordee, penoscrotal transposition, an empty left hemiscrotum, and a palpable right testis. At 2 years and 2 months, his height was 77.5 cm (<−3 SD). Peripheral-blood fluorescence *in situ* hybridization showed mos 47,XYY[108]/46,XY[60]/45,X[32]. Exome sequencing identified no explanatory pathogenic variant, including in *AMH* or *AMHR2*, whereas copy-number analysis detected a *de novo* mosaic Yp11.31–q11.223 duplication concordant with the XYY cell line. The AMH level was >18 ng/mL, the inhibin B level was 166 pg/mL, and testosterone level increased from 0.03 to 4.41 ng/mL after human chorionic gonadotropin stimulation. Bladder distension revealed filling or reflux into a presumed prostatic-utricle-like retrovesical cavity. Computed tomography showed a right intrascrotal testis, a retrovesical cystic lesion, and no identifiable left testis. Combined cystoscopic and laparoscopic assessment revised the diagnosis to a Müllerian remnant complex comprising an infantile uterus, a short vagina, and a left streak-like gonadal-region structure with fallopian-tube-like anatomy. The remnant complex and left gonadal-region tissue were excised with preservation of the right *vas deferens*, and staged hypospadias reconstruction was initiated. Histopathology confirmed fallopian-tube and dysplastic uterovaginal tissues without identifiable gonadal tissue or malignancy. At 2 years and 10 months, second-stage urethroplasty and correction of penoscrotal transposition achieved a straight penis with a glanular meatus. At approximately 7 months after the second-stage procedure, the child had smooth voiding, favorable uroflowmetry, and no urethrocutaneous fistula or urethral stricture.

**Conclusion:**

This case is most appropriately interpreted as 45,X/46,XY/47,XYY mosaic MGD rather than typical 47,XYY syndrome or classic *AMH*-/*AMHR2*-related PMDS. Detectable postnatal AMH does not exclude asymmetric impairment of fetal Müllerian regression. Combined cystoscopic and laparoscopic assessment clarified the anatomy, delineated resection boundaries, and guided individualized staged reconstruction.

## Introduction

Differences/disorders of sex development (DSD) are congenital conditions characterized by atypical chromosomal, gonadal, or anatomical sex development and encompass a heterogeneous group of conditions affecting sex determination and/or differentiation (1, 2). Contemporary practice emphasizes precise characterization of the karyotype, gonadal status, internal duct anatomy, external genital phenotype, and endocrine function within a patient-centered, multidisciplinary framework, rather than reliance on a single diagnostic label (1, 2). This approach is particularly important in sex-chromosome mosaicism, because the proportions of cell lines detected in peripheral blood may not reflect their distribution in gonadal or ductal tissues. Mosaicism involving a 45,X cell line, most commonly 45,X/46,XY mosaicism, is a recognized cause of mixed gonadal dysgenesis (MGD), which is characterized by asymmetric gonadal differentiation, variable undervirilization, hypospadias, asymmetric scrotal development, and, in some cases, persistence of Müllerian structures (3).

Persistent Müllerian duct syndrome (PMDS) is more narrowly defined as persistence of Müllerian derivatives in an otherwise normally virilized 46,XY individual with testes, usually owing to biallelic pathogenic variants in anti-Müllerian hormone (*AMH*) or AMH receptor type 2 (*AMHR2*) (4–6). AMH secreted by fetal Sertoli cells normally induces Müllerian duct regression by approximately the 10th gestational week (6, 7). However, serum AMH concentrations may be normal or elevated in *AMHR2*-related PMDS and in some genetically unresolved cases. Therefore, detectable postnatal AMH does not exclude impaired fetal AMH signaling or incomplete Müllerian regression at the target-tissue level (6, 8, 9). Müllerian remnants have also rarely been described in broader DSD contexts, including complete androgen insensitivity syndrome (10).

The 47,XYY karyotype occurs in approximately 1 in 1,000 male births and is generally associated with a male phenotype (11, 12). Contemporary evidence has replaced the historical “super-male' concept with a more nuanced understanding of variable testicular and reproductive function. Nevertheless, available endocrine evidence predominantly concerns non-mosaic, postpubertal individuals and is only indirectly applicable to young children with mosaic DSD (13). Rare XYY-containing mosaics may present with gonadal dysgenesis and atypical genital development (14, 15), whereas Müllerian remnant-related pathology in individuals with 47,XYY has been reported only exceptionally (16). In this study, we report a male-reared child with 45,X/46,XY/47,XYY mosaicism, severe proximal hypospadias, a left streak-like gonadal-region structure, and a retrovesical Müllerian remnant initially identified as a prostatic-utricle-like cavity during preoperative evaluation. This case illustrates the diagnostic intersection between mosaic MGD and PMDS-like internal anatomy and demonstrates how a combined cystoscopic and laparoscopic assessment can clarify the pelvic anatomy, delineate the extent and resection boundaries of the Müllerian remnant complex, and guide individualized staged reconstruction. This case report was prepared in accordance with the CARE reporting guidelines.

## Case presentation

### Initial presentation and clinical assessment

The patient was a male-reared child born at 39+3 weeks of gestation by cesarean delivery, with a birth length of 50 cm and birth weight of 3,300 g. His parents were non-consanguineous, with no family history of DSD or known hereditary disease. Severe proximal hypospadias with a penile-base meatus, ventral chordee, penoscrotal transposition, an empty left hemiscrotum, and a palpable right testis were noted at birth. Neurodevelopment was age-appropriate.

At a local child-health assessment in February 2024, at approximately 13 months of age, he was assessed as having moderate underweight, stunting, and wasting. At referral on 19 March 2025, aged 2 years and 2 months, his weight was 10.0 kg and height was 77.5 cm (<−3 SD for age and sex). Serial prereferral height measurements and bone-age assessment were unavailable; therefore, prereferral growth velocity and bone-age delay could not be determined.

No Turner-like or other syndromic features were identified. Patent ductus arteriosus and an atrial septal defect had previously been diagnosed, and the ductus had been ligated before urogenital reconstruction. An examination confirmed severe proximal hypospadias with chordee, penoscrotal transposition, absent labioscrotal fusion, a right scrotal testis with hydrocele, and a non-palpable left gonadal region. The External Masculinization Score was 7.5. A chronological summary of the diagnostic evaluation, growth assessment, staged surgical management, and follow-up is provided in Table 1.

**Table 1:** Clinical timeline of diagnostic evaluation, growth assessment, and staged surgical management.

Time point	Clinical event	Essential findings	Management and outcome
At birth	Birth and initial genital findings	Born at 39+3 weeks by cesarean delivery; birth length 50 cm and birth weight 3,300 g. Severe proximal hypospadias with a penile-base meatus, ventral chordee, penoscrotal transposition, an empty left hemiscrotum, and a palpable right testis were noted	The child was reared as male and subsequently underwent multidisciplinary evaluation
February 2024; approximately 13 months	Child-health growth assessment	A local child-health assessment documented moderate underweight, moderate stunting, and moderate wasting	Detailed serial height measurements were not available in the transferred records
12 December 2024; approximately 1 year 11 months	Growth-related endocrine and systemic evaluation	Random growth hormone was 4.87 ng/mL, IGF-1 58.90 ng/mL, and IGFBP-3 3,100 ng/mL. TSH and free thyroxine were within laboratory reference intervals. Routine testing showed no anemia, hypoalbuminemia, renal dysfunction, or major electrolyte abnormality. Age- and sex-specific reference intervals for IGF-1 and IGFBP-3 were unavailable	The growth hormone axis was partially evaluated; no growth hormone stimulation test or bone-age assessment was performed
19 March 2025; age 2 years 2 months	First presentation and multidisciplinary assessment	Weight was 10.0 kg and height 77.5 cm (<−3 SD for age and sex). An examination showed severe proximal hypospadias, chordee, penoscrotal transposition, a right scrotal testis with hydrocele, and a non-palpable left gonadal region; The External Masculinization Score was 7.5. Serial prereferral heights and bone age were unavailable	Multidisciplinary counseling addressed diagnostic uncertainty, sex-of-rearing options, staged reconstruction, fertility and tumor surveillance, growth/endocrine follow-up, and psychosocial support. Male rearing was continued
March 2025	Cytogenetic, genomic, endocrine, and imaging evaluation	Peripheral-blood FISH showed mos 47, XYY [108]/46,XY[60]/45,X[32]. Trio exome sequencing identified no explanatory pathogenic variant, including in AMH or AMHR2. Copy-number analysis showed a *de novo* mosaic Yp11.31–q11.223 duplication. Testosterone levels increased from 0.03 to 4.41 ng/mL after hCG stimulation; AMH was >18 ng/mL and inhibin B 166 pg/mL. Imaging showed a retrovesical cystic lesion, a right testis, and no identifiable left testis	The working diagnoses were severe proximal hypospadias, prostatic utricle, left cryptorchidism, and right hydrocele. A combined cystoscopic and laparoscopic assessment was planned
21 March 2025; age 2 years 2 months	First-stage operation and intraoperative diagnostic revision	Laparoscopy identified an infantile uterus, short vagina, fallopian-tube-like fimbrial tissue, and a left streak-like gonadal-region structure. Cystoscopy showed an opening near the bladder-neck/verumontanum region leading to the vaginal and uterine cavities	The diagnosis was revised to a Müllerian remnant complex associated with asymmetric gonadal dysgenesis. The left gonadal-region and uterovaginal remnants were excised with preservation of the right *vas deferens*. Bilateral hernia ligation, chordee correction, and first-stage Duckett urethroplasty were performed
March 2025	Histopathological evaluation	The left gonadal-region specimen showed fallopian-tube tissue with hydropic change and no identifiable testicular or ovarian tissue. The uterovaginal specimen contained dysplastic infantile uterine and vaginal wall tissues without malignancy	Germ-cell neoplasia marker immunohistochemistry was not performed because no germ-cell component was identified morphologically
10 December 2025; age 2 years 10 months	Second-stage reconstruction	Weight remained 10.0 kg. Cystoscopy showed a patent, non-stenotic urethra and no residual patent communication at the previous Müllerian-remnant opening site, without evidence of infection	The proximal urethral defect was closed, penoscrotal transposition was corrected, and a straight penis with a glanular meatus was achieved
July 2026; approximately age 3 years 6 months	Latest postoperative and growth follow-up	Height was 90 cm and weight 11.0 kg. Height increased by 12.5 cm since March 2025, but precise annualized growth velocity could not be calculated because intermediate measurements were sparse and the exact July date was unavailable. Voiding was smooth; uroflowmetry showed a bell-shaped curve, maximum flow 10 mL/s, voided volume 115 mL, and postvoid residual 6 mL. Ultrasonography showed no recurrent retrovesical mass	No urinary tract infection, fistula, or stricture occurred. Follow-up includes 6-monthly anthropometry and nutritional assessment, baseline bone age at the next endocrinology review, repeat IGF-1/IGFBP-3 with age- and sex-specific interpretation, and growth hormone stimulation testing when indicated, together with urinary, testicular, pubertal, fertility, tumor-risk, and psychosocial surveillance

AMH, anti-Müllerian hormone; AMHR2, anti-Müllerian hormone receptor type 2; FISH, fluorescence *in situ* hybridization; hCG, human chorionic gonadotropin; IGF-1, insulin-like growth factor 1; IGFBP-3, insulin-like growth factor-binding protein 3; SD, standard deviation; TSH, thyroid-stimulating hormone.

### Cytogenetic, genomic, and endocrine evaluation

Peripheral-blood fluorescence *in situ* hybridization (FISH) was performed in 200 interphase nuclei using probes for chromosomes 18, X, and Y. Three sex-chromosome patterns were identified: CSPX×1/CSPY×2 in 108 cells (54%), CSPX×1/CSPY×1 in 60 cells (30%), and CSPX×1 without a detectable Y signal in 32 cells (16%), reported as mos 47,XYY[108]/46,XY[60]/45,X[32].

Trio exome sequencing identified no pathogenic or likely pathogenic variant explaining the DSD phenotype, including no pathogenic variant in *AMH* or *AMHR2*. Copy-number analysis detected a *de novo* mosaic duplication involving Yp11.31–q11.223 (∼21.95 Mb; estimated mosaic fraction, ∼60%), concordant with the XYY cell line. The tissue-specific distribution of the mosaic cell lines was not determined.

Baseline gonadal endocrine testing showed testosterone 0.03 ng/mL, luteinizing hormone 0.99 IU/L, follicle-stimulating hormone 2.47 IU/L, estradiol 5.0 pmol/L, AMH >18 ng/mL, and inhibin B 166 pg/mL. Cortisol and dehydroepiandrosterone levels were within the laboratory reference intervals. Following intramuscular administration of hCG at 1,000 IU daily for 3 consecutive days, testosterone levels increased from 0.03 to 4.41 ng/mL, within the stimulated reference interval of 1.93–8.36 ng/mL. AMH was reported only as >18 ng/mL, whereas the stated pediatric male reference interval was 38.25–332.4 ng/mL. Because dilutional retesting was not performed, the exact concentration was unavailable. Detectable AMH together with inhibin B supported postnatal Sertoli-cell activity but could not determine fetal AMH secretion or target-tissue exposure during Müllerian regression.

A review of previous records identified growth-related endocrine testing performed in December 2024. The random growth hormone level was 4.87 ng/mL, insulin-like growth factor 1 level was 58.90 ng/mL, and insulin-like growth factor-binding protein 3 level was 3,100 ng/mL. Age- and sex-specific reference intervals for IGF-1 and IGFBP-3 were unavailable, and neither growth hormone stimulation testing nor bone-age assessment had been performed. Thyroid-stimulating hormone and free thyroxine levels were within reference intervals, whereas free triiodothyronine level was mildly elevated. Routine hematologic and biochemical testing showed no anemia, hypoalbuminemia, renal dysfunction, major hepatic dysfunction, or clinically significant electrolyte abnormality.

### Imaging and preoperative working diagnosis

Previous urinary tract ultrasonography had not demonstrated a retrovesical cystic lesion. During preoperative evaluation, bladder distension incidentally revealed filling or reflux into a presumed prostatic-utricle-like retrovesical cavity, prompting contrast-enhanced CT of the abdomen and pelvis with three-dimensional reconstruction. MRI was considered but not selected because rapid anatomical clarification was required, sedation would likely have been necessary, and CT was more readily available for simultaneous evaluation of the urinary tract, pelvic cavity, retrovesical lesion, and surrounding structures.

**Figure 1:**
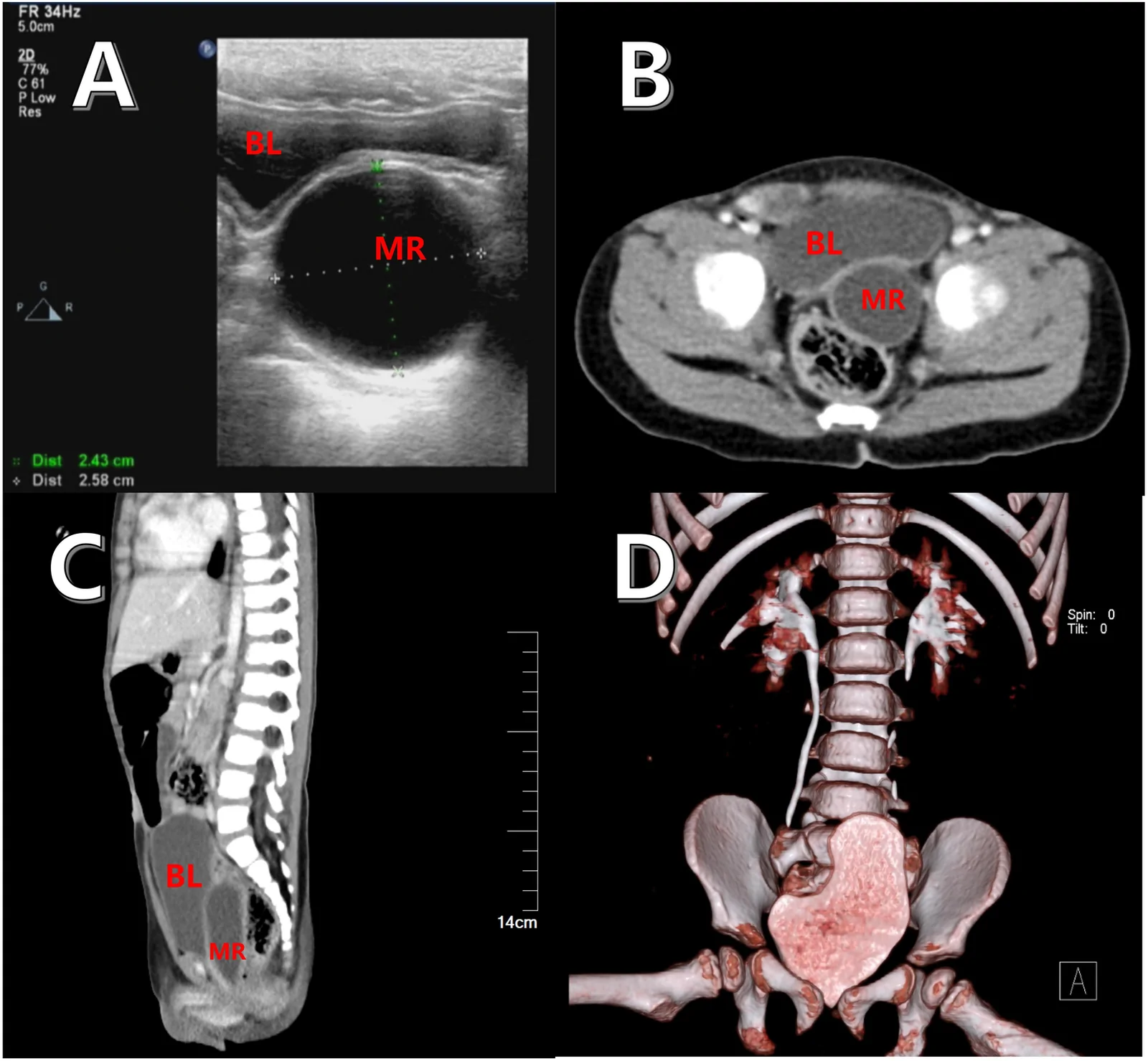
Preoperative imaging of the retrovesical cystic lesion subsequently confirmed as a Müllerian remnant complex. **(A)** Transabdominal ultrasonography shows a well-circumscribed anechoic retrovesical cystic lesion (MR) immediately posterior to the bladder (BL); calipers indicate orthogonal diameters of 2.43 and 2.58 cm. **(B)** Axial contrast-enhanced computed tomography (CT) demonstrates a midline retrovesical cystic lesion (MR) posterior to the bladder (BL). **(C)** Sagittal CT reconstruction shows the lesion (MR) in the midline, posterior and caudal to the bladder (BL), with distal tapering toward the perineum. **(D)** Three-dimensional volume-rendered CT reconstruction of the urinary tract shows no upper urinary tract dilation. BL, bladder; CT, computed tomography; MR, Müllerian remnant.

Targeted ultrasonography showed a right intrascrotal testis with hydrocele, an empty left hemiscrotum, and an anechoic retrovesical structure (Figure 1A). CT demonstrated a well-circumscribed, low-density retrovesical cystic lesion posterior to the bladder and urethra, measuring approximately 28 × 28 mm, without obvious excretory-phase filling. The distal end tapered toward the perineum, suggesting possible communication with the urethra or bladder-neck region. The right testis was visualized, whereas the left testis was not identified, and no upper urinary tract dilation was present (Figures 1B–D). The preoperative working diagnoses were severe proximal hypospadias, a prostatic utricle, left cryptorchidism, and right hydrocele.

Following a multidisciplinary review involving pediatric urology, endocrinology, clinical genetics, radiology, pathology, and perioperative care specialists, the family elected to continue male rearing, consistent with the established sex of rearing and male-predominant phenotype. Counseling addressed the remaining diagnostic uncertainty, alternative sex-of-rearing pathways, the option of deferring definitive genital reconstruction, staged reconstruction, fertility and tumor surveillance, psychosocial support, and long-term follow-up. A combined cystoscopic and laparoscopic assessment was planned to clarify the suspected lower urinary tract communication and define the pelvic anatomy.

### First-stage operation: diagnostic revision and initial reconstruction

**Figure 2:**
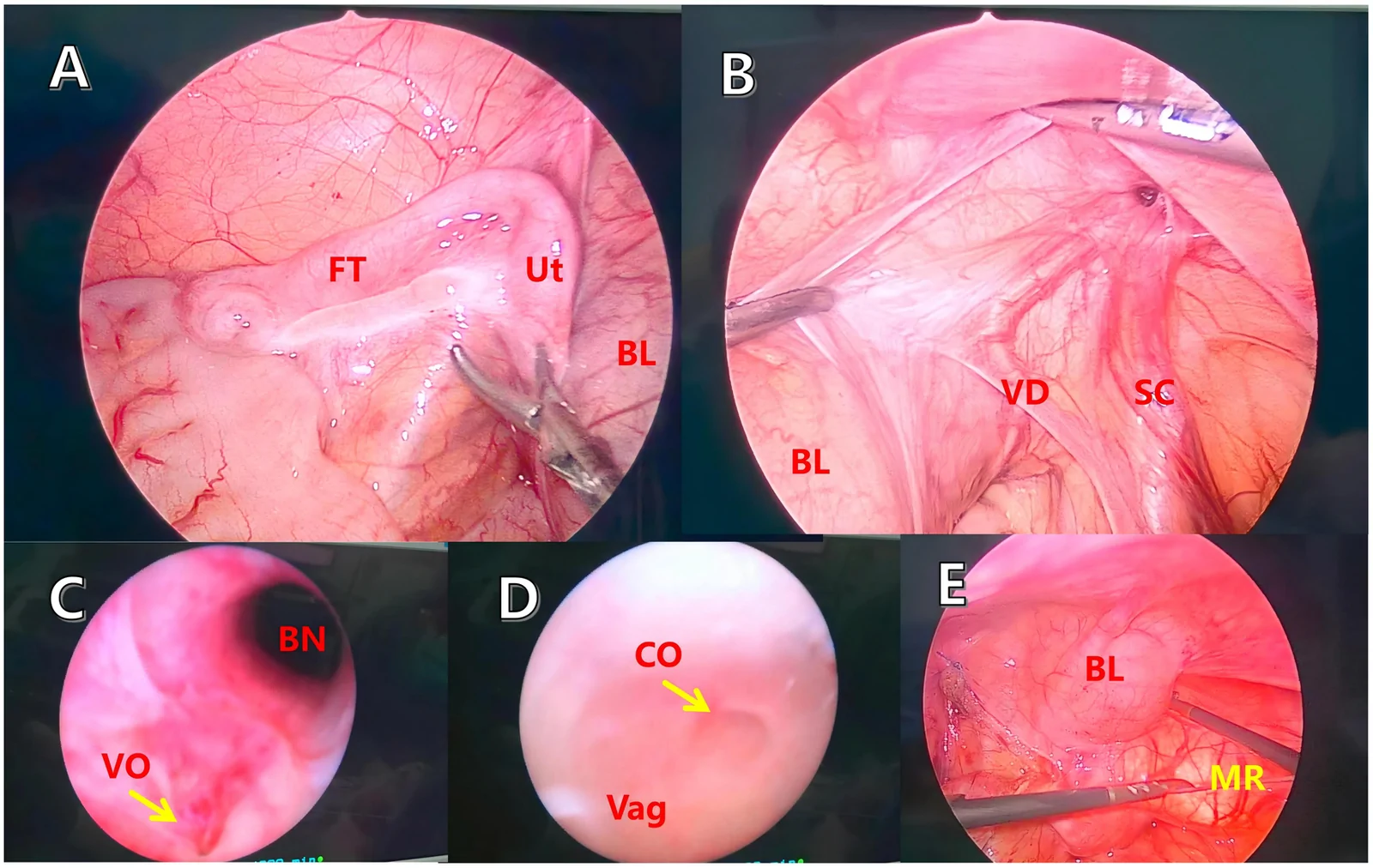
Combined laparoscopic and cystoscopic findings during the first-stage operation. **(A)** Laparoscopy demonstrates the Müllerian remnant complex, including a fallopian-tube-like structure (FT) associated with an infantile uterine remnant (Ut) posterior to the bladder (BL). **(B)** Right-sided laparoscopy shows the *vas deferens* (VD) and spermatic cord (SC) coursing through the internal ring toward the pelvis; these structures were identified and preserved during dissection. **(C)** Cystoscopy shows the opening leading to the vaginal cavity (VO, arrow) near the bladder neck (BN). **(D)** Cystoscopy within the vaginal cavity (Vag) demonstrates the cervical os (CO, arrow). **(E)** Laparoscopy shows the Müllerian remnant complex (MR) posterior to the bladder (BL) during mobilization. The complementary cystoscopic and laparoscopic views delineated the lower urinary tract communication, pelvic extent, anatomical relationships, and resection boundaries of the Müllerian remnant complex while facilitating preservation of adjacent urinary and reproductive structures. BL, bladder; BN, bladder neck; CO, cervical os; FT, fallopian-tube-like structure; MR, Müllerian remnant; SC, spermatic cord; Ut, uterine remnant; Vag, vaginal cavity; VD, *vas deferens*; VO, vaginal opening.

The first-stage operation was performed on 21 March 2025, at the age of 2 years and 2 months. Laparoscopy identified bilateral indirect inguinal hernias, which were treated by high ligation, and prompted revision of the preoperative diagnosis. The left gonadal region contained a streak-like structure with fallopian-tube-like fimbrial anatomy, together with an infantile uterus and a short vaginal cavity, rather than a simple prostatic utricle associated with left cryptorchidism (Figures 2A,E). On the right, the spermatic cord and *vas deferens* traversed the internal ring and were identified and preserved (Figure 2B).

Cystoscopy identified an opening near the bladder-neck/verumontanum region leading into a vaginal cavity measuring approximately 3.0 cm in length, with a visible cervix and uterine cavity. Laparoscopic transillumination confirmed continuity with the Müllerian remnant complex (Figures 2C,D). The combined cystoscopic and laparoscopic findings delineated the lower urinary tract communication, pelvic extent, resection boundary, and relationships of the remnant complex with the bladder neck, urethra, right *vas deferens*, and adjacent urinary structures.

After intraoperative confirmation of the anatomy and in accordance with preoperative parental consent, the left streak-like gonadal-region structure, fallopian-tube-like fimbrial tissue, infantile uterus, and short vaginal remnant were excised, while preserving the right *vas deferens*. Chordee correction and staged Duckett transverse preputial island-flap urethroplasty were then undertaken, with the creation of a planned proximal urethrostomy and coverage using a vascularized dartos pedicle.

### Histopathology

**Figure 3:**
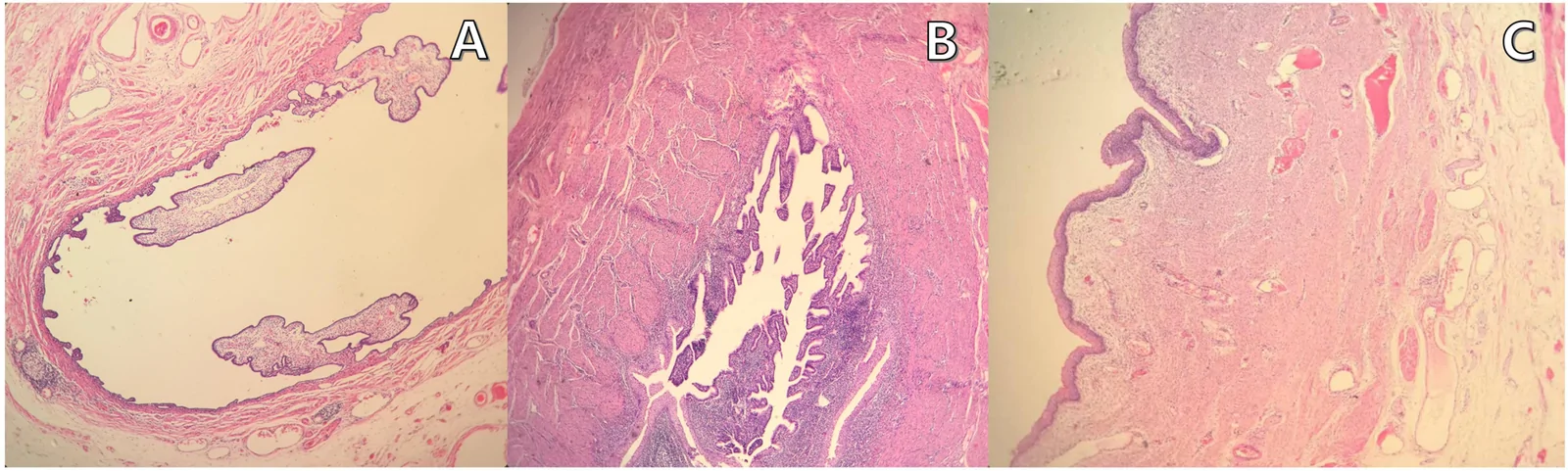
Histopathological findings of the excised specimens. **(A)** The left gonadal-region specimen shows fallopian-tube tissue with plicated mucosa and hydropic change, without identifiable testicular or ovarian tissue. **(B)** The uterovaginal specimen shows epithelium-lined muscular tissue consistent with dysplastic infantile uterine tissue. **(C)** Vaginal wall tissue with overlying epithelium. All panels were stained with hematoxylin and eosin; original magnification, ×100.

A gross examination showed a left streak-like gonadal-region structure associated with fallopian-tube-like fimbrial tissue and infantile uterovaginal tissue. A histopathological examination of the specimen submitted under the clinical designation of “left testis” demonstrated fallopian-tube tissue with hydropic change, without identifiable testicular or ovarian tissue (Figure 3A). The uterovaginal specimen contained dysplastic infantile uterine tissue and vaginal wall tissue, without evidence of malignancy (Figures 3B,C). Because no germ-cell component was identified morphologically, immunohistochemical staining for germ-cell neoplasia markers, including OCT3/4, PLAP, TSPY, and D2-40, was not performed.

### Second-stage operation and follow-up outcomes

The second-stage operation was performed on 10 December 2025, at the age of 2 years and 10 months. Cystoscopy confirmed a patent, non-stenotic urethra and showed no residual patent communication at the previous Müllerian-remnant opening site, without evidence of infection. The residual proximal urethral defect was closed, penoscrotal transposition was corrected, and a straight penis with a glanular meatus was achieved.

Follow-up continued until July 2026, approximately 7 months after the second-stage procedure. The child voided smoothly, with no urinary tract infection, urethrocutaneous fistula, or urethral stricture. Uroflowmetry showed a bell-shaped curve, a maximum flow rate of 10 mL/s, a voided volume of 115 mL, and a postvoid residual volume of 6 mL. Pre- and postvoiding ultrasonography showed no residual or recurrent retrovesical cystic lesion.

The child's weight remained 10.0 kg at the December 2025 admission. By July 2026, his height was 90 cm and weight was 11.0 kg, representing a 12.5-cm increase in height since March 2025. However, because intermediate measurements were sparse and the exact date of the latest measurement was unavailable, a precise annualized growth velocity could not be calculated reliably.

Follow-up was planned every 6 months and included standardized assessment of height, weight, body mass index, nutritional status, and interval growth velocity. Baseline bone-age assessment was planned at the next pediatric endocrinology review, with repeat assessment when clinically indicated. Repeat IGF-1 and IGFBP-3 testing with age- and sex-specific interpretation and growth hormone stimulation testing would be considered clinically indicated. Continued surveillance will also assess urinary function, residual or recurrent Müllerian-remnant-related manifestations, right testicular development, pubertal progression, fertility potential, tumor risk, and psychosocial wellbeing.

## Discussion

This case is best interpreted as MGD in the context of complex sex-chromosome mosaic DSD rather than as classic PMDS or isolated 47,XYY syndrome. The defining features were the convergence of marked undervirilization, 45,X/46,XY/47,XYY mosaicism, asymmetric gonadal development, a preserved right scrotal testis, absence of identifiable testicular or ovarian tissue in the left gonadal-region specimen, histologically confirmed fallopian-tube and uterovaginal remnants, detectable postnatal AMH and inhibin B, and no identified pathogenic variant in *AMH* or *AMHR2*. Classic PMDS is characterized by Müllerian persistence in an otherwise normally virilized 46,XY individual with testes and most commonly results from impaired AMH production or AMHR2-mediated signaling (4–6). The marked undervirilization, complex mosaic karyotype, and asymmetric gonadal findings in this patient are therefore more biologically coherent with mosaic MGD.

The central mechanistic question is why Müllerian-derived structures persisted despite detectable postnatal AMH and measurable inhibin B. Normal Müllerian regression requires appropriately timed fetal Sertoli-cell AMH secretion, AMHR2 expression in Müllerian duct mesenchyme, intact downstream signaling, and adequate local exposure during the critical developmental window (6, 7, 17, 18). A single postnatal AMH measurement, particularly a thresholded rather than fully quantified result, cannot reconstruct fetal AMH secretion, local tissue exposure, or target-tissue responsiveness. This distinction is supported by the PMDS literature: AMH is generally low or undetectable in *AMH*-related PMDS, whereas it may be normal or elevated in *AMHR2*-related PMDS because the primary defect lies in target-tissue responsiveness (4–6, 9). In the present case, AMH >18 ng/mL and inhibin B 166 pg/mL supported postnatal Sertoli-cell activity, most plausibly from the right testis, but did not establish bilaterally intact fetal Sertoli-cell differentiation or AMH action. The left-sided pathological findings therefore support a plausible association between asymmetric gonadal dysgenesis and local impairment of fetal AMH production, exposure, or signaling.

The 45,X/46,XY/47,XYY mosaic karyotype provides a developmental context for this asymmetry. In sex-chromosome mosaicism, cell-line proportions in peripheral blood may differ from those in the gonadal ridges, Sertoli-cell precursors, Wolffian ducts, Müllerian duct mesenchyme, and urogenital sinus derivatives. Peripheral-blood FISH confirmed three cell lines but could not determine their distribution in the right testis, left gonadal-region specimen, or Müllerian-derived tissues. MGD typically involves asymmetric gonadal differentiation, with a testis or dysgenetic testis on one side and a streak or poorly differentiated gonadal region on the other (3, 19). In this patient, the right side contained a scrotal testis, whereas the left gonadal-region specimen contained no identifiable gonadal tissue and was associated with fallopian-tube and uterovaginal structures. The XYY cell line should therefore not be regarded as the sole explanation for Müllerian persistence. A more defensible interpretation is that the 45,X-containing mosaic background contributed to asymmetric gonadal dysgenesis and left-sided failure of normal Müllerian regression.

Y-chromosome-bearing mosaic MGD, retained Müllerian structures, and XYY-containing mosaicism have each been reported (3, 14–16, 19). However, our literature review did not identify a pediatric male-reared patient with the same combination of 45,X/46,XY/47,XYY mosaicism, severe proximal hypospadias, detectable postnatal Sertoli-cell markers, negative *AMH*/*AMHR2* testing, absence of identifiable gonadal tissue in the left gonadal-region specimen, and histologically confirmed fallopian-tube and uterovaginal remnants initially presenting as a prostatic-utricle-like retrovesical cavity. To our knowledge, this case is therefore among the first pediatric descriptions of this specific clinicogenetic, pathological, and surgical constellation.

This case also illustrates a diagnostic pitfall with direct surgical implications. Enlarged prostatic utricles, vagina masculina, posterior urethral cystic lesions, and more extensive Müllerian-derived remnants may have overlapping imaging appearances, particularly in children with severe proximal hypospadias or DSD (20–22). Previous ultrasonography had not demonstrated the retrovesical lesion, which became apparent only when bladder distension revealed filling or reflux into a presumed prostatic-utricle-like cavity. Although contrast-enhanced CT clarified its location and extent, imaging alone could not reliably distinguish a prostatic utricle from vagina masculina, a urethral diverticular lesion, or a more extensive Müllerian remnant complex.

A combined cystoscopic and laparoscopic assessment converted this uncertain radiological impression into a detailed operative map. Cystoscopy defined the lower urinary tract communication near the bladder-neck/verumontanum region, whereas laparoscopy demonstrated the extraluminal relationships among the bladder, uterovaginal remnant, fallopian-tube-like tissue, left gonadal-region structure, right spermatic cord, and right *vas deferens*. This complementary assessment revised the diagnosis, delineated the pelvic extent and resection boundaries of the Müllerian remnant complex, and facilitated preservation of adjacent urinary and reproductive structures.

Surgical management must balance removal of non-functional or potentially harmful tissues against preservation of urinary and reproductive anatomy. Müllerian remnants in PMDS and related DSD conditions may be closely associated with Wolffian derivatives, and aggressive dissection may endanger the *vas deferens* or testicular blood supply. Available surgical evidence supports individualized selection among preservation, division, partial excision, and complete excision according to symptoms, gonadal status, anatomical relationships, the extent of Müllerian development, and malignancy-related concerns (20, 22–26). In this patient, excision of the left streak-like gonadal-region structure and infantile uterovaginal remnants was considered appropriate because the tissues appeared non-functional, no testicular or ovarian component was identified, and continued retention would create anatomical and surveillance concerns. Preservation of the right testis and *vas deferens* was prioritized. The staged reconstructive strategy also allowed gradual correction of severe proximal hypospadias and penoscrotal transposition, while avoiding the complexity of a single extensive procedure.

At approximately 7 months after the second-stage procedure, the child had smooth voiding, no urinary tract infection, urethrocutaneous fistula, or urethral stricture, and favorable uroflowmetry, with a bell-shaped curve, a maximum flow rate of 10 mL/s, a voided volume of 115 mL, and a postvoid residual volume of 6 mL. These findings indicate satisfactory short-term urinary function. Nevertheless, the follow-up period remains insufficient to evaluate late urethral complications, pubertal genital development, fertility potential, or long-term psychosocial outcomes.

Tumor risk should be discussed cautiously. Y-chromosome-bearing mosaic DSD with dysgenetic or intra-abdominal gonadal tissue may be associated with an increased risk of gonadoblastoma or germ-cell neoplasia (3, 27). In our patient, the left gonadal-region remnant was removed and it showed no malignancy; however, the right testis remains *in situ*, and its long-term developmental and neoplastic risk cannot be fully predicted in early childhood. Neoplasia arising in persistent or obstructed Müllerian-derived structures has been reported across different clinical contexts, although such events remain rare (16, 24, 28). Continued surveillance should therefore assess right testicular position, volume, echotexture, pubertal endocrine function, fertility potential, and signs of neoplasia. Reconstructive follow-up should additionally address urethral stricture, diverticulum, fistula, recurrent infection, urinary symptoms, residual or recurrent remnant-related manifestations, and psychosexual development.

Growth evaluation is another long-term management priority. The child had moderate underweight, stunting, and wasting at approximately 13 months of age. At 2 years and 2 months, his height was 77.5 cm (<−3 SD for age and sex) and weight was 10.0 kg. Weight remained 10.0 kg in December 2025 and increased to 11.0 kg by July 2026, when height reached 90 cm. Although height increased by 12.5 cm between March 2025 and July 2026, sparse intermediate measurements and the unavailable exact date of the latest assessment precluded a reliable calculation of annualized growth velocity.

The growth hormone axis had been partially but not comprehensively evaluated. Random growth hormone was 4.87 ng/mL, IGF-1 was 58.90 ng/mL, and IGFBP-3 was 3,100 ng/mL in December 2024. A single random growth hormone concentration cannot establish or exclude growth hormone deficiency because secretion is pulsatile. Moreover, age- and sex-specific reference intervals for IGF-1 and IGFBP-3 were unavailable, and neither growth hormone stimulation testing nor bone-age assessment had been performed. Thyroid-stimulating hormone and free thyroxine were within reference intervals, and routine testing showed no anemia, hypoalbuminemia, renal dysfunction, or major electrolyte abnormality. Nevertheless, nutritional and endocrine contributors to the growth impairment could not be fully distinguished.

Follow-up will include standardized assessment of height, weight, body mass index, nutritional status, and interval growth velocity every 6 months. Baseline bone-age assessment is planned at the next pediatric endocrinology review, with repeat assessment when clinically indicated. IGF-1 and IGFBP-3 will be reassessed using age- and sex-specific reference intervals, and growth hormone stimulation testing will be considered if persistent short stature or subnormal interval growth raises concern for growth hormone deficiency. Any consideration of growth-promoting therapy should be based on serial auxological data, nutritional assessment, bone age, and comprehensive pediatric endocrine evaluation rather than an isolated random growth hormone result.

This report has several limitations. FISH was performed only on peripheral blood, and no cytogenetic analysis was conducted on the right testis, left gonadal-region specimen, or Müllerian-derived tissues; therefore, the proposed tissue-specific mosaic mechanism remains inferential. Postnatal AMH cannot reconstruct fetal secretion or target-tissue exposure during the critical period of Müllerian regression. Although no pathogenic variant in *AMH* or *AMHR2* was identified, non-coding, structural, epigenetic, coverage-related, and tissue-specific abnormalities cannot be completely excluded. Because no germ-cell component was identified morphologically, immunohistochemical staining for germ-cell neoplasia markers was not performed, limiting pathological risk stratification. Growth assessment was limited by sparse longitudinal measurements, absent bone-age assessment, unavailable age- and sex-specific interpretation of IGF-1 and IGFBP-3, and the absence of formal growth hormone stimulation testing. Finally, this is a single case with relatively short follow-up. Asymmetric fetal impairment of AMH production, exposure, or action is a biologically coherent explanation for Müllerian persistence but remains a plausible mechanism rather than a proven causal pathway.

## Conclusion

This male-reared child with 45,X/46,XY/47,XYY mosaicism presented with severe proximal hypospadias, penoscrotal transposition, a left streak-like gonadal-region structure, and a retrovesical Müllerian remnant initially identified as a prostatic-utricle-like cavity during preoperative evaluation. The overall phenotype is more appropriately classified as mosaic mixed gonadal dysgenesis than as typical 47,XYY syndrome or classic *AMH*-/*AMHR2*-related persistent Müllerian duct syndrome. Müllerian persistence most plausibly reflects asymmetric impairment of fetal AMH production, local exposure, or target-tissue signaling associated with left-sided gonadal dysgenesis, whereas the preserved right testis may account for the detectable postnatal AMH and inhibin B levels. A combined cystoscopic and laparoscopic assessment converted an uncertain radiological impression into a detailed operative map, delineated the extent and resection boundaries of the Müllerian remnant complex, and facilitated vas-sparing excision of non-functional Müllerian-derived tissues, preservation of the right testis, and individualized staged reconstruction.

## Patient Perspective

Because the patient was too young to provide his own perspective, this section reflects the views of his legal guardians following multidisciplinary counseling. The family understood and supported continued male rearing, excision of the non-functional Müllerian-derived tissues, preservation of the right testis and *vas deferens*, and the need for long-term follow-up of urinary function, testicular development, growth and endocrine status, pubertal progression, fertility potential, tumor risk, and psychosocial wellbeing. Age-appropriate assent and psychological support will be incorporated into future care as the child matures.

## Data Availability

The datasets presented in this article are not readily available because they contain potentially identifiable clinical and genomic information concerning a minor, and public deposition is not permitted under the informed consent obtained and applicable institutional and ethical restrictions. Requests to access appropriately de-identified data should be directed to Dianyong Liu at cmuldy@163.com and will be considered subject to institutional approval and the scope of the consent obtained.
